# BACE1 across species: a comparison of the *in vivo* consequences of BACE1 deletion in mice and rats

**DOI:** 10.1038/srep44249

**Published:** 2017-03-10

**Authors:** Martin Weber, Tiffany Wu, William J. Meilandt, Sara L. Dominguez, Hilda O. Solanoy, Janice A. Maloney, Hai Ngu, Miriam Baca, Chung Kung, Lisa Lima, Timothy K. Earr, Daniel Fleck, Shannon D. Shields, William F. Forrest, Oded Foreman, Søren Warming, Ryan J. Watts, Kimberly Scearce-Levie

**Affiliations:** 1 Department of Neuroscience, Genentech, 1 DNA Way, South San Francisco, CA 94080, USA; 2 Department of Pathology, Genentech, 1 DNA Way, South San Francisco, CA 94080, USA; 3 Department of Transgenic Technology, Genentech, 1 DNA Way, South San Francisco, CA 94080, USA; 4 Department of Bioinformatics, 1 DNA Way, Genentech, South San Francisco, CA 94080, USA; 5 Department of Molecular Biology, Genentech, 1 DNA Way, South San Francisco, CA 94080, USA

## Abstract

Assessing BACE1 (β-site APP cleaving enzyme 1) knockout mice for general health and neurological function may be useful in predicting risks associated with prolonged pharmacological BACE1 inhibition, a treatment approach currently being developed for Alzheimer’s disease. To determine whether BACE1 deletion-associated effects in mice generalize to another species, we developed a novel *Bace1*^−/−^ rat line using zinc-finger nuclease technology and compared *Bace1*^−/−^ mice and rats with their *Bace1*^+/+^ counterparts. Lack of BACE1 was confirmed in *Bace1*^−/−^ animals from both species. Removal of BACE1 affected startle magnitude, balance beam performance, pain response, and nerve myelination in both species. While both mice and rats lacking BACE1 have shown increased mortality, the increase was smaller and restricted to early developmental stages for rats. *Bace1*^−/−^ mice and rats further differed in body weight, spontaneous locomotor activity, and prepulse inhibition of startle. While the effects of species and genetic background on these phenotypes remain difficult to distinguish, our findings suggest that BACE1’s role in myelination and some sensorimotor functions is consistent between mice and rats and may be conserved in other species. Other phenotypes differ between these models, suggesting that some effects of BACE1 inhibition vary with the biological context (e.g. species or background strain).

Alzheimer’s disease (AD) is characterized by neurofibrillary tangles and amyloid plaques in the brain, with hyperphosphorylated tau and amyloid beta (Aβ) peptides as their key components, respectively. Sequential proteolytic cleavage of membrane-bound amyloid precursor protein (APP) generates soluble Aβ peptides that, according to the amyloid cascade hypothesis of AD^1^, accumulate in the brain and lead to other downstream pathologies including neuronal loss, and ultimately AD. The first cleavage step is largely due to the activity of the aspartyl protease BACE1 (β-site APP cleavage enzyme 1), which is widely expressed in the brain (see expression patterns on biogps.org). This suggests that inhibition of BACE1 could prevent or reduce the accumulation of Aβ in the brain, reducing AD-related pathology and possibly functional impairments. Strong support for this view comes from human genetics with mutations in APP or presenilin, one of the four subunits of γ–secretase responsible for the final cleavage step towards Aβ, leading to an overproduction of Aβ and causing AD (cf.^2^,^3^,^4^). Specifically, a mutation at the BACE1 cleavage site in APP results in more efficient cleavage and causes familial early onset AD^5^. However, a protective mutation in APP (A673T) has been shown to reduce cleavage and production of Aβ and correspondingly decreased risk for AD^6^. These and other data have built a well-established rationale for a role of BACE1 in the pathophysiology of AD and as a potential target for the treatment of AD.

Not surprisingly, inhibitors of BACE1 are currently being developed for the treatment of AD^3^,^7^,^8^, and show promise as therapeutics in preclinical and clinical studies. However, putative detrimental effects of prolonged BACE1 inhibition are less well understood. Studies suggest that there are many different BACE1 substrates, increasing chances of on-target side effects^3^,^4^,^9^,^10^,^11^. Substrates linked to detrimental effects of BACE1 inhibition or deletion include neuregulin 1 (NRG1; specifically NRG1 type III), close homolog of L1 (CHL1), and others^4^. Yet, given the abundance of BACE1 substrates, predicting potential liabilities of prolonged BACE1 inhibition in a bottom-up manner from the substrates is not practically feasible. An alternative approach is to take advantage of *Bace1*^−/−^ animals.

Several phenotypes have been reported in BACE1 null mice. For example, increased mortality and reduced body weight has been observed in mice with constitutive inactivation of the gene encoding BACE1^12^,^13^. Savonenko *et al*.^14^ demonstrated that *Bace1*^−/−^ mice display several changes in schizophrenia-related preclinical measures, most prominently deficits in prepulse inhibition of startle^15^. Two studies^16^,^17^ reported a reduction in nerve myelination and altered bundling of small caliber axons in BACE1 null mice that were due to lack of BACE1-mediated NRG1 type III processing. Changes in nerve myelination can affect a wide range of behaviors from sensation to motor function, and both reduced grip strength and increased pain sensitivity in mice lacking BACE1 have been linked to changes in myelination^17^. Others^18^ discovered alterations in motor function in *Bace1*^−/−^ mice using gait patterns as the primary readout. These findings were linked to deficits in muscle spindle formation and maintenance. BACE1 has also been implicated in axon guidance of olfactory sensory neurons and the formation of the olfactory bulb^19^,^20^,^21^, possibly mediated via the BACE1 substrate CHL1^21^. While this suggests that BACE1 mutant mice might also have deficits in olfaction, no functional study has been carried out to date.

Most reports are based on *Bace1*^−/−^ mice and there is only one published report of a BACE1 knockout rat model^22^. In this study, Fielden *et al*.^22^ showed that an ocular phenotype involving thinning of the retina in *Bace1*^−/−^ mice^23^ is not observed in *Bace1*^−/−^ rats. While Fielden *et al*.^22^ focused on ocular readouts, this raises the more general question of how well other findings in BACE1 null mice may translate to rats and possibly other species, or if they are unique to the mouse species or specific strain in a given study. To address this question, we studied phenotypes in *Bace1*^−/−^ rats and mice in parallel.

## Materials and Methods

### Animals

All animal experiments were approved by the Genentech IACUC and comply with the Institute for Lab Animals’ guidelines for the humane care and use of laboratory animals. Animals were housed on a 14 h light/10 h dark cycle with *ad libitum* access to water and food. *Bace1*^−/−^ rats were generated by SAGE Labs using zinc-finger nuclease (ZFN) technology^24^ to create a 137-base pair deletion spanning the translation initiation start site in exon 1 of the rat *Bace1* gene, corresponding to chr8:48,766,315–48,766,452 (RGSC 5.0/rn5 assembly). Rats were generated and maintained on a Sprague Dawley (Taconic) background. *Bace1*^−/−^ mice^25^ were originally obtained from Jackson labs (B6.129-*Bace1*^*tm1Pcw*^/J) and were maintained on a C57BL/6J genetic background at Genentech. Note that the *Bace1*^−/−^ rat and mouse models differ with regard to their respective background strains, which are outbred and inbred, respectively. Heterozygous matings generated both *Bace1*^−/−^ experimental animals and *Bace1*^+/+^ littermate controls.

### Behavioral tests

#### General

Animals were counterbalanced by genotype to avoid systematic effects of recording chambers, arenas, or time of day; acclimated for at least 20 min prior to the beginning of the experiment; and tested during the light phase, with room lights on. An experimenter blinded to genotype performed behavioral scoring.

#### Locomotor activity

Rats: grey thermoplastic cages (40.0 (W) × 40.0 (L) × 34.5 (H) cm) were arranged underneath a video camera linked to a video tracking system. Distance travelled was recorded. Mice: transparent thermoplastic cages (40.5 (W) × 40.5 (L) × 38 (H) cm) were fitted with infrared (IR) beams (3 cm above floor) were used to record horizontal beam breaks^26^. A total of 30 min of locomotor activity beginning with the placement of the animals into the locomotor chambers were analyzed.

#### Acoustic startle

Startle testing was conducted according to established rat and mouse protocols as described previously^26^,^27^,^28^. Animals were placed in the startle chambers for a 5 min acclimation period. Background noise was 70 (rats) or 65 (mice) dB(A). One hundred (rats) or sixty-five (mice) 1-millisecond readings were collected for each animal beginning at the onset of the startle stimulus and averaged to define startle magnitude. The session began and ended with a block of four 40-ms, 120-dB(A) noise bursts (pulse-alone trials). Between these blocks the following trial types were presented in pseudorandom order: 16 pulse-alone trials; a pulse-alone preceded 100 ms (onset-to-onset) by a 20-ms noise burst of either low, medium, or high prepulse intensity (10 trials each). The low, middle and high prepulse intensity was 5, 10 or 15 (rats) or 4, 8, 16 dB above background (mice)^27^,^28^,^29^,^30^. Inter-trial interval (ITI) between active trials ranged from 8 to 22 s and averaged 15 s. Total session duration was 18.5 min.

#### Odor habituation test

Animals were placed in empty transparent thermoplastic cages (mice: 28 (L) × 18 (W) × 13 (H) cm; rats: 43 (L) × 22 (W) × 21 (H) cm) for a 20 min acclimation period during which a water-soaked cotton-swab was suspended above the floor. After the acclimation period, a fresh cotton-swab soaked in one of three odorants was presented (vanilla extract, Simply Organic^®^, Frontier Natural Products, Norway, IA, USA, 1:1000 in water; limonene, 183164, Sigma-Aldrich, St. Louis, MO, USA, 1:1000 in water, or rosemary oil, Now^®^ Foods, Bloomingdale, IL, USA, 1:2000 in water). Seven successive trials of three minutes each were conducted. Each trial began by presenting a fresh cotton swab and the time spent touching, licking, or sniffing within 1 cm from the tip of the swab was recorded. The sequence of odors was arranged to allow odor habituation as well as dishabituation: trials 1–3: odor A; trials 4–6: odor B; trial 7: odor C. The sequence of odor presentations was counterbalanced between animals. Seven rats (4 *Bace1*^+/+^, 3 *Bace1*^−/−^) and six mice (5 *Bace1*^+/+^, 1 *Bace1*^−/−^) that did not engage in the task (two or fewer investigations of the cotton swabs throughout the seven trials) were excluded from analyses and graphs. For analysis of odor habituation the investigation times during trials 1 and 4 were averaged and compared to the average investigation time during trials 3 and 6. For analysis of odor dishabituation the average investigation time during trials 3 and 6 was compared to the average investigation time from trials 4 and 7.

#### Hot-plate test

A hot plate apparatus for rats and mice set to 55 °C was used^31^,^32^,^33^. Each animal was placed onto the hot plate. The time until the animal licked a hind paw, jumped, or reached the cut-off time (30 s), whichever came first, was recorded, then the animal was removed from the plate. There were 5 min intervals between trials. Three trials were averaged per animal.

#### Balance beam

Wooden dowels (1.04 m long) of varying diameter (~3.2 cm for rats, 2.5, 1.9 and 1.1 cm for mice) were placed horizontally between two platforms ~50–65 cm above the ground. A plastic shelter (rats: cylinder 16 cm (H), 16 cm in diameter; mice: box 14 (L) × 9 (W) × 7.5 (H) cm) was located on a target platform, slightly higher than the starting platform, creating a slight inclined path on the beam towards the shelter. Animals were acclimated to the experimental procedure. On the test days, animals were placed onto the beam facing toward (rats) or away from (mice) the shelter. Rats were tested in two trials on the same beam. Mice were tested in three trials on each size beam. The number of foot slips made while covering a distance of 80 cm toward the shelter was counted and averaged per trial for each animal^34^.

#### Tissue collection

Animals were deeply anesthetized and whole blood was collected via cardiac puncture and placed in plasma collection tubes with EDTA. Cerebrospinal fluid (CSF) was collected from the cisterna magna, placed in 20 μl of 10% BSA in sterile water and immediately stored on dry ice. After PBS perfusion, the brain was collected and the left hippocampus and cortex were frozen and homogenized for Western blotting (see below), and the right hippocampus and cortex were frozen and homogenized in 10 volumes of 5 M Guanidine HCL in 50 mM Tris (pH 8.0) and further diluted 1:10 in Casein Blocking Buffer (0.25% casein, 0.05% sodium azide, aprotinin (20 mg/ml), 5 mM EDTA (pH 8.0), leupeptin (10 mg/ml) in PBS) for Aβ40 analysis.

Sciatic nerves were harvested proximal to the branch point of the tibial and peroneal nerves and fixed overnight in 4% PFA. Nerves were post-fixed in half Karnovsky’s fixative: 2% paraformaldehyde, 2.5% glutaraldehyde in 0.1 M cacodylate buffer, for 24 hours. Nerves were washed in 0.1 M Sorensen’s buffer, post-fixed with agitation in 1% osmium tetroxide in 0.1 M Sorensen’s buffer for 24 hours, at 4 °C and washed again in 0.1 M Sorensen’s buffer. They were incubated en bloc in 2% uranyl acetate for 1 hour and washed again in 0.1 M Sorensen’s buffer. Sciatic nerves were dehydrated with agitation at 70%, 95%, and 100% in 200 proof ethanol twice for 10 minutes, and agitated in propylene oxide twice for 10 minutes. The nerves were then infiltrated with eponate 12 resin (Ted Pella, Redding, CA, USA) with agitation as follows: 1:1 eponate/propylene oxide for 12 hours, followed by 2:1 eponate/propylene oxide for 4 hours followed by pure eponate for 4 hours. The nerves were embedded in eponate in flat silicone embedding molds and polymerized in eponate at 70 °C overnight. Blocks were sectioned on an automated ultramicrotome set to cut 1 μm at a 6 degree knife angle at 1.60 mm/sec. Sections were collected on Superfrost positively charged glass slides (Thermo Scientific, Kalamazoo, MI, USA). Slides were stained with 2% p-phenylenediamine in 50% ethanol for 15 minutes, rinsed in water for 10 minutes, air dried, and coverslipped with Cytoseal 60 (Thermo Scientific, Kalamazoo, MI, USA).

#### Aβ ELISAs

Plasma, CSF, and brain tissues (cortex or hippocampus) were collected and processed for Aβ40 measurement by ELISA as previously described^35^,^36^,^37^. Briefly, rabbit polyclonal antibody specific for the carboxy-terminus of Aβ40 (Millipore, Bedford, MA, USA) was coated onto plates, and biotinylated anti-mouse Aβ monoclonal antibody M3.2 (Covance, Dedham, MA, USA) was used for detection. The assay had a lower limit of quantification of 1.96 pg/ml in plasma and 39.1 pg/g in brain.

#### Western Blots

Tissue was weighed and homogenized in 10 volumes of buffer (1% NP40, or RIPA with Roche Phosphatase and Complete, EDTA-free, protease inhibitor cocktail tablets) using a Qiagen TissueLyser II (2 × 3 min, 30 Hz). Samples were rotated for 30 minutes at 4 °C then centrifuged at a relative centrifugal force (RCF) of 20,817 × g for 30 minutes at 4 °C. Supernatants were collected and protein concentrations were determined by BCA. 20 μg of protein were separated on a NuPage 10% Bis-Tris PAGE gel and transferred to nitrocellulose membrane. Blots were incubated with the monoclonal rabbit BACE1 antibody (D10E5, 1:1000, Cell Signaling, Danvers, MA, USA), GAPDH (14C10, 1:1000, Cell Signaling), or β-actin (A5316, 1:1000, Sigma-Aldrich, St. Louis, MO, USA). Densitometric analysis was performed. Samples were normalized against loading control (GAPDH or Actin), and were then normalized to control *Bace1*^+/+^ samples (set to 1.0).

#### Myelin quantification

Digital images of p-phenylenediamine stained sciatic nerve cross-sections were acquired using a whole slide scanning system at x400 magnification with a resolution of 0.23 microns/pixel. Regions of interest (ROI) outlining the cross-sectional area of nerve bundles were manually drawn and analyzed at full resolution using Matlab 8.1 (Mathworks, Natick, MA, USA). The images were converted to gray scale and initial segmentation of lumen and myelin area was determined using intensity thresholding and rolling-ball filtering. Additional segmentation was performed by applying the Laplace operator on Gaussian smoothed images, and by morphological and HSI (hue, saturation, intensity) processing within watershed separated regions. Detected objects were further selected based on morphological reconstruction, mean intensity, size and shape factor analysis. Together this led to the detection of an estimated 80% or more of the axons per ROI. Data of one to three cross-sections per animal were averaged. For each animal ≥4000 axons were sampled to determine precise estimates of the following parameters: axonal density, average myelin and lumen area per myelinated axon, and average g-ratio (d_inner_/d_outer_). The average myelin and lumen areas per myelinated axon were summed up to yield the total area of the axon with its surrounding myelin sheath (A_outer_). We determined approximate g-ratios by deriving the inner diameter (d_inner_) and outer diameter (d_outer_) from the axon lumen area (A_inner_) and axon plus myelin area (A_outer_), respectively. Assuming circular shape of axon-plus myelin cross-section, the formula d = 2 * (A/π)^0.5^ was used, where d corresponds to d_inner_, or d_outer_, and A to A_inner_ or A_outer_, respectively. This led to a single average value per animal for each readout, which was then used for statistical analyses. Image analysis was performed blinded to genotype.

#### Statistical Analysis

Data were analyzed using mixed-model or between-subjects ANOVAs as appropriate, followed by post-hoc comparisons using Tukey’s HSD to account for more than two comparisons between factors or factor levels. Mortality analyses were based on >4,000 mice and 2,000 rats from our BACE1 breeding colonies. These data were assessed via Kaplan-Meier survival estimates followed by generalized Wilcoxon Chi-Square (χ^2^) tests for the estimated survival functions between genotypes. The day of euthanasia is considered the date the censoring criterion was met. Censoring means that the documented survival of the animal until a specific date (the censoring date) is used for the analyses, but no assumptions as to how long the animal may have lived after the censoring date are being made. A second set of analyses using an alternative approach to account for health alerts and subsequent euthanasia in animals was conducted and is outlined in the supplements (Figure S1). The day an animal was found dead or not found in the cage (if of weaning age) was considered as the day of its death. For both sets of analyses, censoring criteria was also met on the day that an animal was a) submitted for transfer out of the breeding colony and no information was available as to whether an animal was alive or not, or b) alive on the last day that the database had been updated (4/4/2016) prior to the database query (4/7/2016). Line or bar graphs with mean ± SEM values are shown. A 2-tailed alpha of 0.05 was used. *p < 0.05, **p < 0.01, ***p < 0.0001.

## Results

An overview of the results is shown in Table 1.

### Aβ40 protein levels in brain and plasma are reduced in *Bace1* ^−/−^ animals

**Figure 1:**
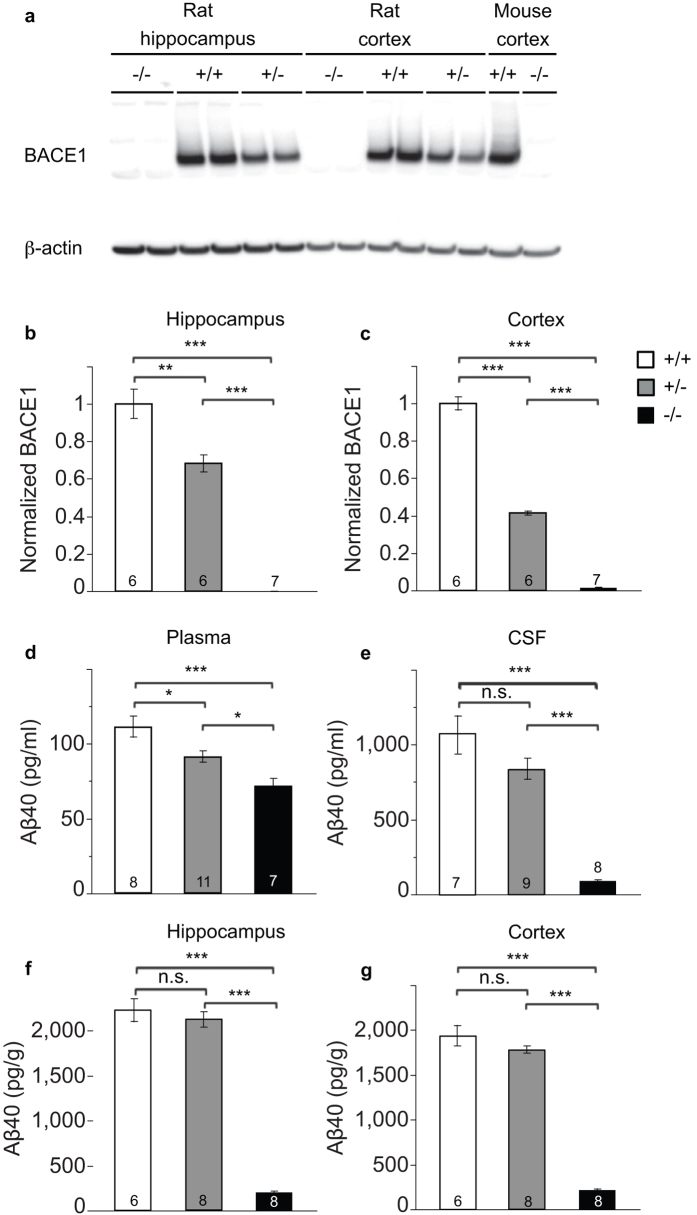
Effects of BACE1 deletion on peripheral and brain Aβ40 levels in rats. **(a)** BACE1 protein loss was confirmed by Western blotting in the hippocampus and cortex of *Bace1*^+/+^, *Bace1*^+/−^, and *Bace1*^−/−^ rats. Cortical extracts from wildtype and *Bace1*^−/−^ mice shown right. Densitometric analysis of BACE1 immunoreactivity normalized to β-actin and wildtype rats for the rat hippocampus **(b)** and cortex **(c)**. Aβ40 concentrations in *Bace1*^−/−^ (white bars) and *Bace1*^+/−^ rats (grey bars) compared with *Bace1*^+/+^ controls (black bars) in plasma **(d)**, CSF **(e)** hippocampus **(f)** and cortex **(g)**. *p < 0.05, **p < 0.01, ***p < 0.0001. Values are mean ± SEM, numbers represent sample size (number of rats, n).

To confirm the absence of BACE1 protein and examine the resulting impact on Aβ production in rats, we measured BACE1 protein levels and Aβ concentrations in a novel BACE1 knockout rat generated using zinc-finger nuclease technology (ZFN)^24^. BACE1 protein was undetectable by Western blot rat *Bace1*^−/−^ cortex and hippocampus. Cortex from *Bace1*^−/−^ and *Bace1*^+/+^ mice was used as negative and positive control, respectively (Fig. 1a). Quantitative comparisons of BACE1 protein levels in *Bace1*^+/+^, *Bace1*^+/−^, and *Bace1*^−/−^ rats demonstrated gene-dose dependent effects of BACE1 deletion on BACE1 protein levels (Rat hippocampus: Fig. 1b, ANOVA effect of genotype: F(2, 16) = 114.28 (p < 0.0001); +/+ > −/− (p < 0.0001); +/+ > +/− (p = 0.001); +/− > −/− (p < 0.0001); Rat cortex: Fig. 1c, ANOVA effect of genotype: F(2, 16) = 595.89 (p < 0.0001); +/+ > +/− > −/− (p < 0.0001 between all genotypes). There was a significant reduction in plasma Aβ40 concentrations in *Bace1*^−/−^ (−35%) and *Bace1*^+/−^ (−18%) rats relative to *Bace1*^+/+^ controls (Fig. 1d). Aβ40 concentrations in CSF (Fig. 1e), hippocampus (Fig. 1f), and cortex (Fig. 1g) were significantly reduced by ~90% in the BACE1 knockout rats compared with BACE1 wildtype controls. Notably, relatively minor reductions in Aβ40 were observed in the CNS of *Bace1*^+/−^ rats (CSF: −21%; hippocampus: −4%; cortex: −8%), suggesting that ~50% of endogenous BACE1 is sufficient to cleave most of the APP under physiological conditions (Plasma: F(2, 23) = 12.37 (p = 0.0002); +/+ > −/− (p < 0.0001); +/+ > +/− (p = 0.027); +/− > −/− (p = 0.039); CSF: F(2, 21) = 40.26 (p < 0.0001); +/+ > +/− vs. −/− (p < 0.0001); +/+ vs. +/− (n.s); hippocampus: F(2, 19) = 211.97 (p < 0.0001); +/+ > −/− (p < 0.0001); +/+ vs. +/− (n.s.); +/− > −/−, (p < 0.0001); cortex: F(2, 19) = 261.63 (p < 0.0001); +/+ > −/− (p < 0.0001); +/+ vs. +/− (n.s); +/− > −/− (p < 0.0001)).

### Effects of *Bace1* deletion on mortality in mice and rats

**Figure 2:**
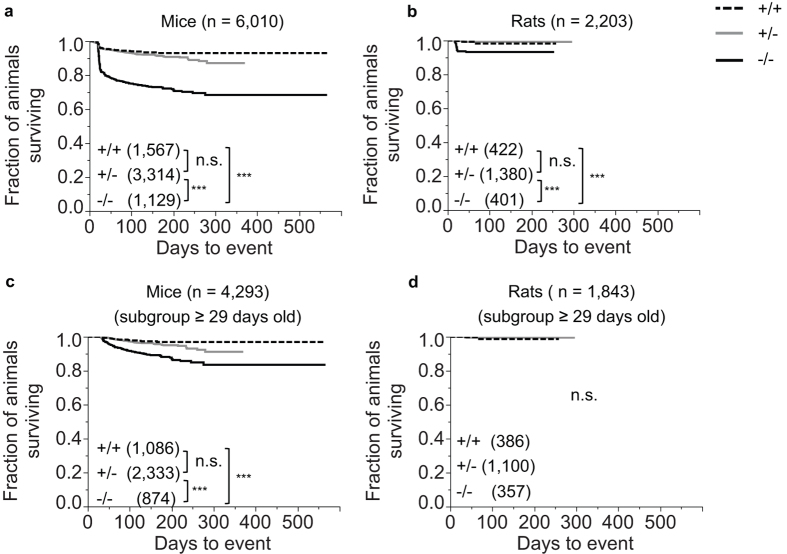
Effects of BACE1 deletion on survival. *Bace1*^−/−^ mice (**a**) and rats (**b**) showed significantly increased mortality relative to heterozygous and wildtype animals. Since a major fraction of the mortality occurred before or at weaning age, subgroup analyses were carried out for animals that lived beyond weaning (≥29 days; (**c**,**d**). In these subgroups, *Bace1*^−/−^ mice continued to have increased mortality when compared to *Bace1*^+/+^ mice (**c**), while the corresponding subgroup of *Bace1*^−/−^ rats were indistinguishable from their wildtype counterparts (**d**). Censoring marks not shown. ***p < 0.0001. Values shown represent the fraction of animals surviving. Numbers represent sample size (number of animals, n).

Previous studies have shown that mortality is increased in BACE1 null mice relative to their wildtype counterparts^12^. Here, mortality analyses from a total of 6,010 mice (1567 *Bace1*^+/+^, 3,314 *Bace1*^+/−^, and 1,129 *Bace1*^−/−^) revealed higher mortality in BACE1 null mice when compared to wildtype as well as heterozygous mice, while no significant differences were found between wildtype and heterozygous mice (χ^2^ (2) = 309.06 (p < 0.0001); +/+ < −/−, χ^2^ (1) = 163.82 (p < 0.0001); +/− < −/−, χ^2^ (1) = 239.37 (p < 0.0001); +/+ vs. +/−, χ^2^ (1) = 1.08 (p = 0.299), Fig. 2a).

Corresponding analyses from 2203 rats (422 *Bace1*^+/+^, 1380 *Bace1*^+/−^, and 401 *Bace1*^−/−^) also revealed increased mortality in BACE1 null rats when compared to wildtype and heterozygous rats, while no significant differences were found between *Bace1*^+/+^ and *Bace1*^+/−^ rats (χ^2^ (2) = 67.08 (p < 0.0001); +/+ < −/− (χ^2^ (1) = 18.56 (p < 0.0001)); +/− < −/− (χ^2^ (1) = 56.70 (p < 0.0001)); +/+ vs +/− rats (χ^2^ (1) = 0.24 (p = 0.62)), Fig. 2b). It is notable that while *Bace1*^−/−^ mortality was significantly increased in both species, mortality appeared to be generally higher in *Bace1*^−/−^ mice compared to *Bace1*^−/−^ rats (Fig. 2a and b).

Since mortality was most pronounced in juvenile animals before weaning, subgroup analyses were conducted to assess if mortality in BACE1 null animals persists after weaning. Animals that were dead or censored before the age of 29 days were excluded and the analyses were repeated. For mice, this subgroup contained 4,293 animals in total (1,086 *Bace1*^+/+^, 2,333 *Bace1*^+/−^, 874 *Bace1*^−/−^). Increased mortality in BACE1 null mice relative to wildtype and heterozygous mice was again detected, while *Bace1*^+/+^ were not significantly different from *Bace1*^+/−^ mice (χ^2^ (2) = 74.90 (p < 0.0001)); +/+ < −/− (χ^2^ (1) = 41.84 (p < 0.0001)); +/− < −/− (χ^2^ (1) = 55.24, (p < 0.0001)); +/+vs. +/− (χ^2^ (1) = 1.09 (p = 0.30)), Fig. 2c).

In rats, no genotype effect was detected in the corresponding subgroup containing 1,843 animals in total (386+/+, 1,100+/−, 357 −/−; χ^2^ (2) = 0.08, (p = 0.96), Fig. 2d).

Additional analyses were conducted using contrasting model assumptions with respect to documented health alerts in the database (see Supplements, Statistical analysis section) leading to similar conclusions: Overall mortality is a) increased in both BACE1 null mice and rats relative to wildtype and heterozygous animals; b) not significantly different between wildtype and heterozygous animals in both species; c) most pronounced in juvenile *Bace1*^−/−^ mice and rats; and d) increased post-weaning in *Bace1*^−/−^ mice, but not in *Bace1*^−/−^ rats.

### Body weight is reduced in adult *Bace1* ^−/−^ mice, but normal in adult *Bace1* ^−/−^ rats

**Figure 3:**
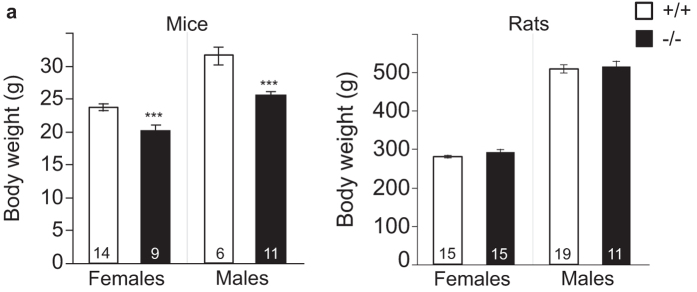
Effects of BACE1 deletion on body weight. **(a)** Both male and female *Bace1*^−/−^ mice showed significantly reduced body weights relative to wildtype controls. **(b)** Both male and female *Bace1*^−/−^ rats had similar body weights relative to *Bace1*^+/+^ controls. Mice and rats were ~20 weeks old when weights were taken. ***p < 0.0001. Values are mean ± SEM, numbers represent sample size (number of animals, n).

Body weight of ~20 week old mice is reduced in *Bace1*^−/−^ mice (Fig. 3a), confirming previous studies^12^,^13^. *Bace1*^−/−^ rats, however, did not show any reduction in body weight at the same age (Fig. 3b). For mice, analysis of body weight revealed a main effect of genotype, with lower body weights for −/− vs. +/+ animals; and the expected main effect for sex, and with no interaction effect of genotype and sex (genotype: F(1, 26) = 37.60, (p < 0.0001); sex: F(1, 26) = 71.73 (p < 0.0001); genotype × sex: F(1, 26) = 2.33 (p = 0.14)).

In contrast, for rats, there was no main effect of genotype on body weight. The expected sex effect was observed, and there was no interaction of genotype and sex (genotype: F(1, 56) = 0.87, (p = 0.35); sex: F(1, 56) = 149.95 (p < 0.0001); genotype × sex: F(1, 56) = 0.09 (p = 0.763), Fig. 3b).

While we do not have body weight data on pre-weaning animals, we did mine our animal breeding databases for pre-weaning animals that were flagged as runts (according to visual inspection by experienced colony managers). There were numerically higher fractions of *Bace1*^−/−^ runts when compared to their respective control groups in both species (Supplementary Table S1).

### Locomotor activity is increased in *Bace1* ^−/−^ mice, but reduced in *Bace1* ^−/−^ rats

**Figure 4:**
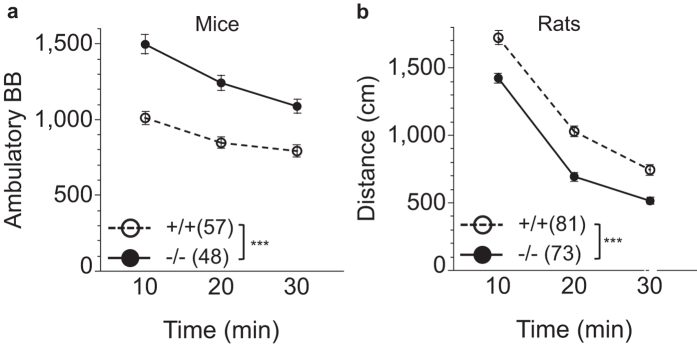
Effects of BACE1 deletion on locomotor activity. **(a)** Locomotor activity was significantly increased in BACE1 null mice, but significantly reduced in BACE1 null rats **(b)** across all time points relative to wildtype controls. ***p < 0.0001. Values are expressed as mean ± SEM, numbers represent sample size (number of animals, n).

Consistent with other reports^12^,^14^ we observed increased locomotor activity in BACE1 null mice compared to wildtype mice (Fig. 4a). Unexpectedly, a *decrease* in locomotor activity was observed in BACE1 null rats when compared to wildtype rats (Fig. 4b).

ANOVA analysis of locomotor activity in mice revealed increased locomotor activity in −/− vs. +/+ mice. As expected, locomotor activity in mice increased over time. There also was a genotype by time interaction reflecting mildly lower differences in activity levels between the genotypes over time (genotype: F(1, 103) = 41.97 (p < 0.0001); time: F(2, 206) = 88.75 (p < 0.0001); genotype × time: F(2, 206) = 7.86 (p = 0.0005)).

Similar analysis in rats revealed a main effect of genotype with lower locomotor activity in −/− vs. +/+ rats, and the expected effect of time (genotype: F(1, 152) = 35.98, (p < 0.0001); time: F(2, 304) = 910.94 (p < 0.0001); genotype × time: F(2, 304) = 2.68 (p = 0.071)).

Taken together *Bace1*^−/−^ mice and rats display distinct locomotor behavior, with pronounced hyperlocomotion in *Bace1*^−/−^ mice, but pronounced hypolocomotion in *Bace1*^−/−^ rats.

### Startle magnitude is reduced in both *Bace1* ^−/−^ mice and rats. Prepulse inhibition of startle is reduced in *Bace1* ^−/−^ mice and increased in *Bace1* ^−/−^ rats

**Figure 5:**
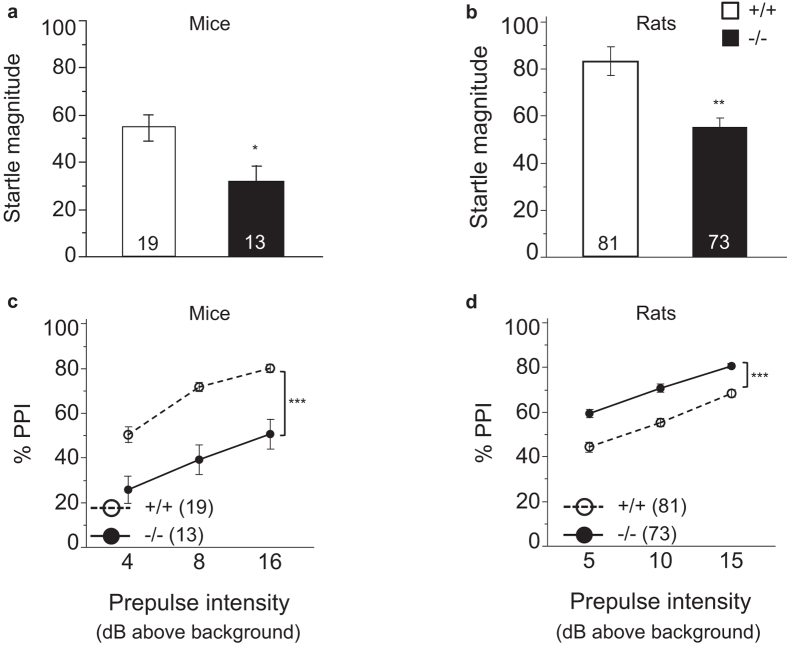
Effects of BACE1 deletion on startle magnitude and % PPI. (**a,b**) Startle magnitude was significantly reduced in *Bace1*^−/−^ mice and rats relative to *Bace1*^+/+^ controls. **(c,d)** % PPI was significantly reduced in *Bace1*^−/−^ mice, but significantly enhanced in *Bace1*^−/−^ rats relative to wildtype animals. *p < 0.05, **p < 0.01, ***p < 0.0001. Values are expressed as mean ± SEM, numbers represent sample size (number of animals, n).

BACE1 null and wildtype animals were compared in measures of startle magnitude and PPI^15^,^27^,^28^,^29^,^30^,^38^. We find reduced startle magnitudes in *Bace1*^−/−^ mice (Fig. 5a) and rats (Fig. 5b) relative to their *Bace1*^+/+^ controls. This indicates functionally analogous changes with respect to the startle circuitry in BACE1 null mice and rats relative to wildtype animals (Genotype: mice: F(1, 30) = 7.01 (p = 0.013; rats: F(1, 152) = 14.55, p = 0.0002)).

Overall % PPI in mice was reduced in *Bace1*^−/−^ mice (Fig. 5c) as indicated by ANOVA analysis. As expected, increased prepulse intensities resulted in greater % PPI, but this was independent of genotype (genotype: F(1, 30) = 37.40, (p < 0.0001); prepulse intensity: F(2, 60) = 33.26 (p < 0.0001); genotype × prepulse intensity: F(2, 60) = 0.69 (p = 0.50)).

In rats, the results were reversed, with increased % PPI in *Bace1*^−/−^ rats (Fig. 5d). There was a main effect of prepulse intensity with greater % PPI with increasing prepulse intensities, but no interaction of genotype by prepulse intensity (genotype: F(1, 152) = 39.67 (p < 0.0001)); prepulse intensity: F(2, 304) = 217.52, (p < 0.0001); genotype × prepulse intensity: F(2, 304 = 1.21 (p = 0.298)).

A change in the basic startle circuitry is predicted to affect startle magnitude. This kind of altered processing of the prepulse or pulse, however, may also affect % PPI and yield a false positive PPI finding, given that these changes are independent of the PPI circuitry in the forebrain. To assess if the genotype effect on startle magnitude in the present study impacts the PPI measure, separate analyses were carried out for subsets of animals generated by eliminating the extreme responders until the effects of genotype on startle magnitude were numerically balanced across genotypes. These analyses showed similar results as when the full sets of animals were included, and demonstrate that the genotype effect on startle magnitude is unlikely to account for the genotype effect on % PPI for either mice or rats (see Supplements; Figure S2). Taken together, these observations indicate functionally analogous changes with respect to the startle circuitry in BACE1 knockout mice and rats relative to wildtype animals, but differences in the impact of BACE1 removal on the circuitry underlying sensorimotor gating in mice and rats.

### Odor habituation and dishabituation are normal in BACE1 null mice and rats

**Figure 6:**
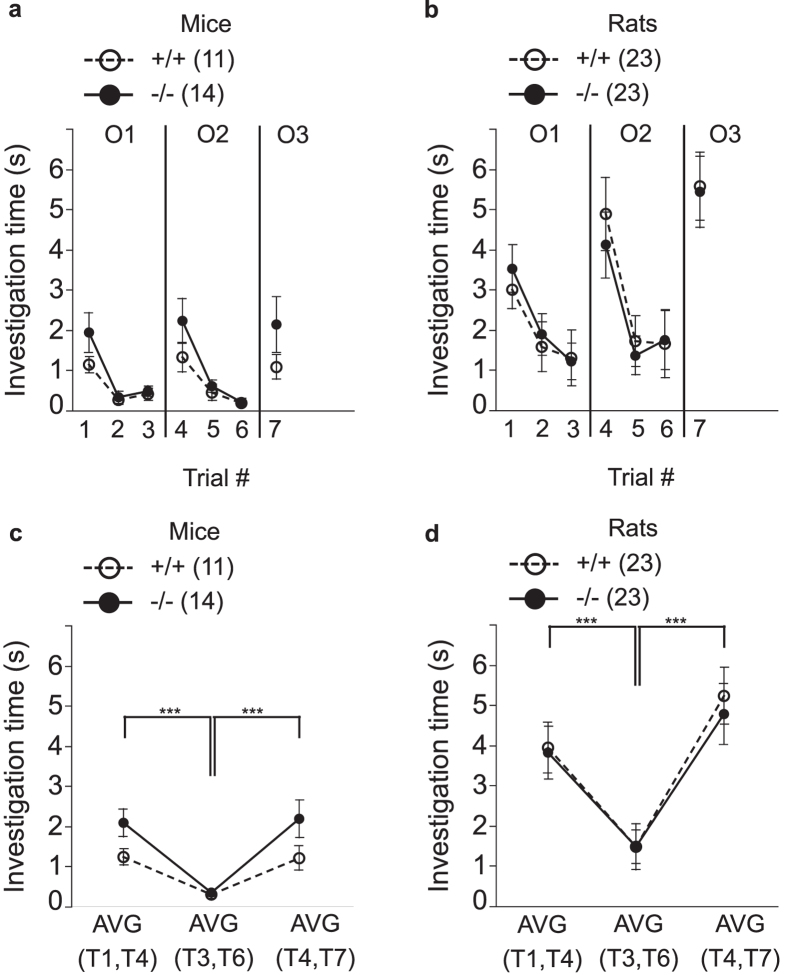
Effects of BACE1 deletion on odor habituation and dishabituation. **(a,b)** The odor investigation time (s) during a total of 7 trials across three different odors (O1, O2, O3). Odor habituation manifests as a reduction in investigation time with repeated presentations of the same odor (trial 1 to 3 for O1 and trial 4 to 6 for O2). Odor dishabituation manifests in an increased investigation time from the last presentation of a repeatedly encountered odor to the first presentation of a novel odor (trial 3 vs. 4, and trial 6 vs. 7). **(c,d)** For data analyses for odor habituation, investigation times of the first presentation of O1 and O2 were averaged (i.e. average of trial 1 and 4 (AVG(T1,T4))) and compared to the average of the last presentations of O1 and O2 (i.e. average of trial 3 and 6 (AVG(T3,T6))). For data analyses for odor dishabituation, investigation times for the last presentation of O1 and O2 were averaged (i.e. average of trial 3 and 6 (AVG(T3,6))) and compared to the average of the first presentation of the subsequently presented novel odors O2 and O3 (i.e. average of trial 4 and 7 (AVG(T4,T7))). Odor habituation and dishabituation were apparent in all groups, without a main effect of genotype for either species. ***p < 0.0001. Values are expressed as mean ± SEM, numbers represent sample size (number of animals, n).

Previous studies have demonstrated a role for BACE1 in axon guidance of olfactory neurons and in the formation of the olfactory bulb^19^,^20^,^21^. To assess olfactory function, we compared *Bace1*^−/−^ and *Bace1*^+/+^ animals in measures of olfactory habituation and dishabituation. Surprisingly, olfactory habituation and dishabituation appeared normal in both *Bace1*^−/−^ mice and rats, indicating intact gross olfactory function (Fig. 6a,b). For statistical analysis, habituation and dishabituation scores were calculated for each animal for each odor set (Fig. 6c,d). For habituation in mice, ANOVA showed the expected main effect of trial number, with reduced investigation times for the third presentations of the same odor when compared to the first presentation. There was a non-significant trend towards a genotype effect with more investigation times in *Bace1*^−/−^ vs. *Bace1*^+/+^ mice, and a trend towards a genotype by trial number effect. Analysis of the odor dishabituation data for mice demonstrated the expected main effect of trial number, with reduced investigation times for the third presentations of the odor when compared to the very first presentation of a novel odor. There was no significant genotype effect and no genotype by trial number interaction effect (Odor habituation: genotype: F(1, 23) = 3.265 (p = 0.083); trial number: F(1, 23) = 42.31 (p < 0.0001); genotype × trial number: F(1, 23) = 3.82 (p = 0.063); odor dishabituation: genotype: F(1, 23) = 2.64 (p = 0.12); trial number: F(1, 23) = 21.74 (p < 0.0001): genotype × trial number: F(1, 23) = 2.51 (p = 0.13), Fig. 6c).

Analysis of the odor habituation data for rats revealed the expected main effect of trial number, with reduced investigation times for the third presentations of the same odor when compared to the first presentation. There was no significant genotype effect, and no genotype by trial number interaction effect. Analysis of the odor dishabituation data for rats revealed the expected main effect of trial number, with reduced investigation times for the third presentations of an odor when compared to the first presentation of a novel odor. There was no significant genotype effect, and no genotype by trial number interaction effect (odor habituation: genotype: F(1, 44) = 0.01 (p = 0.94); trial number: F(1, 44) = 42.35, (p < 0.0001); genotype × trial number: F(1, 44) = 0.03 (p = 0.87); odor dishabituation: genotype: F(1, 44) = 0.08 (p = 0.77); trial number: F(1, 44) = 67.46 (p < 0.0001); genotype × trial number: F(1, 44) = 0.27 (p = 0.60), Fig. 6d).

These data show that a lack of BACE1 does not impair odor habitation or dishabituation in either mice or rats. On the contrary, exploration times of novel odors tended to be enhanced in *Bace1*^−/−^ mice.

### Hot plate response latency is increased in *Bace1* ^−/−^ mice and rats

**Figure 7:**
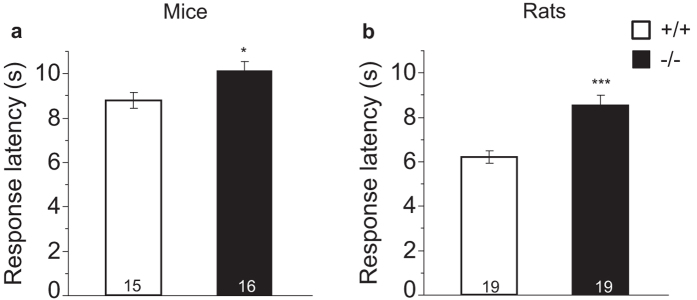
Effects of BACE1 deletion on hotplate response latencies. Latency (s) to respond to a noxious heat stimulus (55 °C hot plate) was significantly longer in both *Bace1*^−/−^ mice **(a)** and rats **(b)** relative to *Bace1*^+/+^ animals. *p < 0.05, ***p < 0.0001. Values are expressed as mean ± SEM, numbers represent sample size (number of animals, n).

A previous study showed reduced latencies for *Bace1*^−/−^ mice in the hot plate test of response to noxious heat^17^. Surprisingly, we observed increased latencies to respond to noxious heat in both *Bace1*^−/−^ mice and rats when compared to their *Bace1*^+/+^ counterparts indicating a reduction in nociception in *Bace1*^−/−^ animals: ANOVA of the response latency demonstrated main effects of genotype (mice: F(1, 29) = 6.32 (p = 0.018); rats: F(1, 36) = 18.87, p < 0.0001, Fig. 7a,b).

### Balance beam foot slips are increased in both *Bace1* ^−/−^ mice and rats

**Figure 8:**
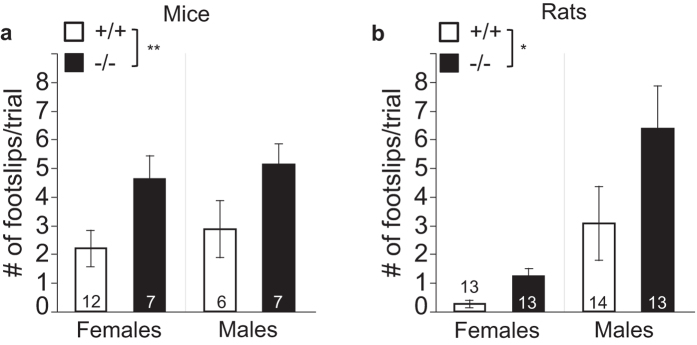
Effects of BACE1 deletion on foot slips in a balance beam task. Across both sexes, *Bace1*^−/−^ mice **(a)** and rats **(b)** showed significantly increased numbers of foot slips relative to wildtype controls. *p < 0.05, **p < 0.01. Values are expressed as mean ± SEM, numbers represent sample size (number of animals, n).

Previous studies have demonstrated gait abnormalities in *Bace1*^−/−^ mice^18^, prompting us to assess BACE1 null mice and rats in a balance beam task. The number of foot slips was increased in both *Bace1*^−/−^ mice and rats suggesting that BACE1 removal impairs this aspect of motor function. Since, in rats, a large fraction of the variance in this measure is linked to sex, two-way ANOVAs with the factors genotype and sex were conducted: ANOVA for mice revealed a main effect of genotype, no main effect of sex, and no interaction of genotype with sex (genotype: F(1, 28) = 8.61 (p = 0.0066); sex: F(1, 28) = 0.54 (p = 0.47); F(1, 28) = 0.001 (p = 0.92), Fig. 8a). Similar analyses for rats showed a main effect of genotype with more foot slips in −/− rats, a main effect of sex, but no interaction of genotype with sex (genotype: F(1, 48) = 4.62 (p = 0.037); sex: F(1, 48) = 16.03 (p < 0.0001); genotype × sex: F(1, 48) = 1.39, (p = 0.24), Fig. 8b).

### Myelination deficits in *Bace1* ^−/−^ mice and rats

**Figure 9:**
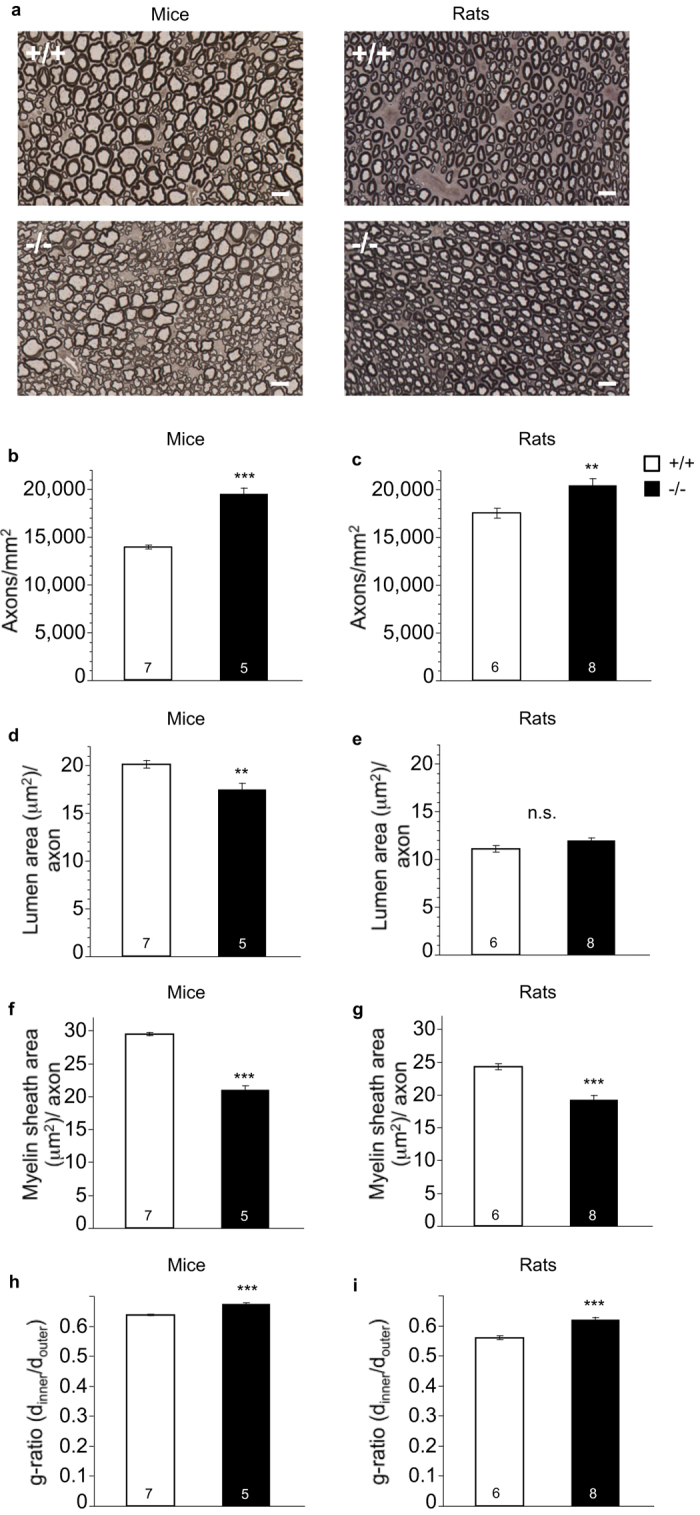
Effects of BACE1 deletion on sciatic nerve anatomy. **(a)** Example images of p-phenylenediamine myelin staining in *Bace1*^−/−^ mice and rats and *Bace1*^+/+^ controls. Myelin-positive regions stain dark. Axon density (axons per mm^2^) was enhanced in *Bace1*^−/−^ mice and rats. **(b,c)**. The average lumen area (in μm^2^) of axons was reduced in *Bace1*^−/−^ mice, but not in *Bace1*^−/−^ rats relative to *Bace1*^+/+^ controls **(d,e)**. The average area of the myelin sheath (in μm^2^) surrounding axons was reduced in *Bace1*^−/−^ mice and rats relative to *Bace1*^*+/+*^ controls **(f,g)**. The g-ratio (d_inner_/d_outer_) of the lumen diameter (d_inner_) relative to the diameter of the lumen and its surrounding myelin sheath (d_outer_) was increased in BACE1 −/− mice and rats **(h,i)**. **p < 0.01, ***p < 0.0001. Values are expressed as mean ± SEM, numbers represent the number of animals analyzed for each measure. The scale bar corresponds to 10 μm.

Previous studies have described myelination deficits in *Bace1*^−/−^ mice^16^,^17^. We found that, with the exception of the axon lumen area, both *Bace1*^−/−^ mice and rats have very similar alterations in nerve anatomy when compared to wildtype controls: Axon density in cross-sections of the sciatic nerve was significantly increased in *Bace1*^−/−^ mice (Fig. 9b) and rats (Fig. 9c). Next we assessed two measures that are likely to affect conduction velocity^39^,^40^,^41^ namely the cross-sectional area of the axon lumen and the overall myelination as measured by quantifying the area of the myelin sheath surrounding the axon: The area of the axon lumen was reduced in *Bace1*^−/−^ mice (Fig. 9d), but was not significantly altered in *Bace1*^−/−^ rats (Fig. 9e), while the area of the myelin sheath surrounding the axon was lower in both *Bace1*^−/−^ mice (Fig. 9f) and *Bace1*^−/−^ rats (Fig. 9g) relative to control animals. Next, the g-ratio, a widely used indicator of axon myelination that relates the lumen diameter to the diameter of the lumen plus myelin sheath was calculated. g-ratios were significantly increased in BACE1 null mice (Fig. 9h) and rats (Fig. 9i) relative to control animals, indicative of relatively thinner myelin sheaths in knockout animals from both species (axon density: mice: F(1, 11) = 106.08 (p < 0.0001), rats: F(1, 12) = 9.70 (p = 0.0089); axon lumen: mice: F(1, 11) = 12.90 (p = 0.0042), rats: F(1, 12) = 4.02 (p = 0.06); myelin sheath area: mice: F(1, 11) = 202.35 (p < 0.0001), rats: F(1, 12) = 32.67 (p < 0.0001); g-ratio: mice: F(1, 11) = 85.12, p < 0.0001, rats: F(1, 12) = 30.75 (p < 0.0001)).

These results suggest that *Bace1*^−/−^ animals have pronounced alterations in axonal anatomy of the sciatic nerve, apparent on multiple levels of analysis relative to *Bace1*^+/+^ controls. In particular, lack of BACE1 leads to increased axon density and significantly thinner myelin sheaths around axons in the sciatic nerves of both mice and rats.

## Discussion

We generated and characterized a novel rat *Bace1*^−/−^ model and directly compared it with an established *Bace1*^−/−^ mouse model, focusing on behaviors and endpoints that are likely to be modulated by BACE1 activity. This cross-species characterization can highlight potential safety liabilities that may emerge after prolonged pharmacological inhibition of BACE1. Similar BACE1 null phenotypes across both species included changes in sciatic nerve anatomy and several measures of sensorimotor function. In contrast, several other phenotypes of BACE1 null animals differed strongly between the two species, including spontaneous locomotor activity levels, prepulse inhibition of startle, and indicators of general health including body weight and premature mortality in post-weaning animals. Our findings are summarized in Table 1.

In our biochemical analysis, BACE1 null rats were similar to BACE1 null mice^42^,^43^: BACE1 protein was virtually absent in the brains of *Bace1*^−/−^ mice and rats alike confirming the completeness of the knockout. While 90% of the Aβ40 was absent in the CNS (CSF, hippocampus and cortex) of *Bace1*^−/−^ rats, a significant amount of Aβ40 was still present in plasma suggesting a role of alternative processing pathways. While BACE1 heterozygous rats had ~40–70% of wildtype BACE1 protein levels, BACE1 haploinsufficiency only had a mild impact on Aβ40 levels in the CNS, consistent with studies in mice^44^. This suggests that in rodents, endogenous BACE1 levels are well above the levels necessary for APP processing. These findings demonstrate that the biochemical consequences of BACE1 deletion, at least with respect to APP processing, are consistent between rats and mice^42^,^43^,^44^,^45^ and in alignment with observations in humans following treatment with BACE1 inhibitors^46^,^47^,^48^,^49^, suggesting that this rat model has adequate predictive validity as a translational model in these measures.

We used our breeding colony databases to conduct analyses of the effects of BACE1 deletion on mortality. Two alternative models were calculated to differentially account for health alerts in the databases. While the two models, by design, arrive at different survival curves, the statistical analyses led to similar conclusions: Mortality is increased in *Bace1*^−/−^ mice, confirming earlier findings in mice^12^. While increased mortality was also detected in *Bace1*^−/−^ rats relative to *Bace1*^+/+^ and *Bace1*^+/−^ animals, the extent of premature mortality in rats was less marked than in *Bace1*^−/−^ mice. It is also notable that heterozygous animals of both species showed no increase in mortality. This suggests that in normal animals, BACE1 may be present in excess of physiologically important substrates – a hypothesis that is consistent with our observations of minimal Aβ40 reductions in heterozygous rats. Mortality in both *Bace1*^−/−^ rats and mice was most pronounced close to weaning age, suggesting a role for BACE1 in development on this measure. A second set of analyses in the subpopulation of post-weaning animals revealed that mortality in older *Bace1*^−/−^ animals, while less drastic, continues to be enhanced in knockout mice, but not in knockout rats. Note that the sample size for rats (n = 1,843), while still substantial, is smaller than for mice (n = 4,293), thus the likelihood to detect very small survival differences, may be somewhat reduced. Furthermore, rats were not maintained beyond the age of 300 days.

Body weight, another indication of general health, was reduced in adult *Bace1*^−/−^ mice confirming earlier studies^12^,^13^. Surprisingly, this effect was absent in adult *Bace1*^−/−^ rats. In both species, however, there was a numerically higher fraction of runts at pre-weaning age among Bace1^−/−^ animals. This finding may indicate an increased likelihood of poor general health in *Bace1*^−/−^ rats and mice at this developmental stage. The relatively increased mortality of Bace1^−/−^ rats and mice of this age group corroborates this view. However, we cannot ascertain that the discrepancies in body weight and mortality phenotype between adult Bace1^−/−^ rats and mice are truly due to differences between these species. It is also conceivable that the higher genetic diversity of our outbred strain *Bace1*^−/−^ rat model (Sprague-Dawley) in part masks the higher mortality and the body weight phenotype observed in adult *Bace1*^−/−^ mice, which are on an inbred background strain (C57BL/6J).

We further confirmed that locomotor activity in a novel environment is increased in *Bace1*^−/−^ mice relative to *Bace1*^+/+^ mice^12^,^14^. Again surprisingly, the opposite phenotype of reduced activity was observed in BACE1 null rats, suggesting that this difference must also be due to additional factors that differ between the two models.

Startle magnitude and PPI were examined next: We found that startle magnitude, a sensorimotor measure that reflects the activity of the basic startle reflex^15^,^30^,^38^, was reduced in both *Bace1*^−/−^ mice and rats. PPI on the other hand reflects the activity of diverse forebrain regions that leads to an inhibition of the basic startle response. PPI is also impaired in several neuropsychiatric disorders^15^,^30^,^38^. Savonenko *et al*.^14^ have reported reduced PPI in *Bace1*^−/−^ mice, an observation that we confirmed here. The rat PPI phenotype was again distinct from the mouse phenotype, with PPI in *Bace1*^−/−^ rats enhanced relative to *Bace1*^+/+^ rats. The higher baseline PPI value in BACE1 control mice, when compared to control rats, may favor detecting PPI reductions in BACE1 null mice and PPI increases in null rats. However, % PPI in *Bace1*^−/−^ mice was less than in *Bace1*^+/+^ rats, while % PPI in *Bace1*^−/−^ rats was at or above the % PPI observed in *Bace1*^+/+^ mice, ruling out that the dynamic range of our instrumentation was insufficient to detect bidirectional changes in behavior. While studies testing the effects of genetic manipulations on PPI in mice have been performed for many years, this study is one of the first to evaluate the effect of a gene deletion on PPI in rats. As more genetically modified rats become available, future studies can further dissect species differences in the contribution of specific genes and biological pathways to this translationally relevant behavior.

Previous studies in BACE1 null mice discovered changes in axon guidance of olfactory sensory neurons and the formation of the olfactory bulb^19^,^20^,^21^, yet functional studies in BACE1 null animals have been lacking. Here, no evidence of functional impairment was detected as both BACE1 null mice and BACE1 null rats displayed normal olfactory habituation and dishabituation. While this does not exclude the possibility of subtle differences, our data do not support the hypothesis that olfaction is grossly impaired following genetic deletion of BACE1. By extension, prolonged treatment with BACE1 inhibitors is unlikely to result in major on-target disruption of olfaction.

Hu *et al*.^17^ reported reduced escape latencies to noxious heat stimuli in BACE1 null mice, but we found that response latencies in a similar hotplate assay were prolonged in both *Bace1*^−/−^ mice and rats. One difference is that Hu *et al*.^17^ used hind limb flicks as an additional criterion that constitutes a pain response. This may account for the lower overall response latencies seen in null and wildtype mice in their data when compared to our mouse data. Hu *et al*.^17^ did not report locomotor activity data in their study, but the replicated observation that *Bace1*^−/−^ mice are hyperactive^12^,^13^ raises the possibility that hyperactivity may confound the observed pain withdrawal latencies. We detected increased pain withdrawal latencies in both *Bace1*^−/−^ mice and rats, despite their opposite locomotor phenotypes, indicating that genotype-dependent effects on locomotor activity are unlikely to confound hot plate measures in this study.

Sensorimotor circuits like acoustic startle or pain withdrawal could be affected by changes in the way neural signals are conveyed to and from the periphery. For example, nerve axons with larger cross-sectional lumen area (or diameter) and thicker myelin sheaths tend to have increased conduction velocity^39^,^40^,^41^. Therefore, we examined the type of changes in peripheral nerve myelination and axonal size that were previously reported in *Bace1*^−/−^ mice^16^,^17^. Using an automated detection method, data from several thousand myelinated axons per animal were quantified. This led to the detection of several changes in nerve anatomy with very high consistency between animals of the same experimental group (see SEM values in Fig. 9b–i). Note that the number of animals (not axons) is the unit of observation. We found increased axon density in sciatic nerve bundles in *Bace1*^−/−^ mice and rats. The cross-sectional axon lumina were reduced in *Bace1*^−/−^ mice, but there was no significant difference between *Bace1*^−/−^ rats and *Bace1*^+/+^ rats in this measure. The area of the myelin sheath around axons was reduced in *Bace1*^−/−^ mice and rats, corresponding to increased g-ratios (d_inner_/d_outer_)^16^,^17^, suggesting relatively thinner myelin sheaths in knockout animals from both species. These findings lead to the testable hypothesis that the conduction velocity in axons of BACE1 null animals is reduced. However, whether or not such changes are causally related to functional changes in sensorimotor function remains unclear.

Since *in vivo* data from inducible BACE1 knockout animals have not been published to date, it also remains difficult to determine if BACE1-dependent changes in myelination occur exclusively during development, or if they could also occur if BACE1 activity was reduced or inhibited in adults^3^,^50^. An alternative approach to investigate the role of BACE1 inhibition on myelination and sensorimotor function in adults is chronic treatment with BACE1 inhibitors. Cheret *et al*.^18^ used both genetic and pharmacological approaches to link BACE1 function to maintenance of gait, and the formation and maintenance of proprioceptive muscle spindles. It is possible that changes in nerve myelination could also affect complex motor behavior, including the gait abnormalities described by Cheret *et al*.^18^ and the balance beam deficits we observed in both BACE1 null mice and rats. However, a recent report^49^ indicated no overt myelination deficits were observed in rats following 6 months of daily treatment with the BACE1 inhibitor MK-8931. While no detailed quantification of nerve myelination was presented in their study, this would be in line with the hypothesis that the nerve myelination deficits observed in BACE1 null animals primarily reflect developmental deficits. Taken together, further studies comparing the effect of genetic vs. pharmacological inhibition of BACE1 on motor function, nerve anatomy, and muscle spindle maintenance are ultimately needed to determine if BACE1 removal primarily affects motor behavior through developmental pathways or through pathways that are active in the mature nervous system. Ongoing clinical observations of motor behaviors in patients treated with BACE1 inhibitors will also provide essential insight into the role of BACE1 in motor behaviors in humans.

While genetic deletions can help predict safety liabilities that may emerge from on-target effects of BACE1 inhibitors, the life-long absence of BACE1 in *Bace1*^−/−^ animals remains different from prolonged pharmacological treatments, in part because of off-target effects that are inherent in most pharmacological treatments. Furthermore, constitutive gene deletion models lack the gene throughout development, while pharmacological treatment approaches for AD are expected to begin later in life. Therefore studies of this type might detect developmental phenotypes that will not translate to chronic pharmacological inhibition in the adult. Conditional *Bace1*^−/−^ animals that lack BACE1 during adulthood but not during development are much needed in order to minimize the likelihood of this possibility. On the flip side, constitutive mouse and knockout rat models may offer the advantage of increased sensitivity for prediction of on-target effects of decade-long pharmacological BACE1 inhibition in humans that may be missed in time-limited pharmacological inhibition in rodents. Furthermore, the “drop-out” due to mortality in *Bace1*^−/−^ animals raises the concern that the fraction of animals that survived until the completion of the experiments represents a subset of animals that is less affected by BACE1 deletion.

The present study highlights the fact that there may be important species-dependent differences in the function of specific genes, even in biologically conserved pathways. Until recently, genetic studies have been limited to mice, but the rat is the typical species used for certain type of studies in drug discovery, including microdialysis and notably toxicology. This has made it difficult to determine if effects observed in these studies are on-target or not. Conceivably, the ability to do the type of phenotypic analyses reported here in rats that lack expression of a relevant drug target could support better design of such studies and more definitive interpretation of results. Indeed, if the question arises if a toxicology finding in rats is on-target or not, the definitive proof that the finding is off-target would be a persistence of that finding in knockout animals dosed with the same drug. To date only one published study on BACE1 null animals examined rats^22^. Yet, cross-species *in vivo* studies are urgently needed since BACE1 inhibition is an important therapeutic approach for AD in humans^3^,^4^. Careful characterization of *in vivo* phenotypes in animals with genetic BACE1 deletion provides a way to predict liabilities of pharmacological BACE1 inhibition in the clinic, in particular if animals from multiple species are tested. Using such *in vivo* data as a starting point, it may be possible to identify the biological system and ultimately the BACE1 substrates that drive specific phenotypes in a top-down manner. While beyond the scope of the present work, one approach to guide such studies may be RNA sequencing in rat and mouse models of BACE1 inhibition, to detect convergent transcriptome differences in both mice and rats lacking BACE1.

Remarkably, the present study has identified several BACE1-related phenotypes with strong cross-species concordance despite the many differences between the mouse and rat models, ranging from genetic to environmental. These phenotypes include measures of nerve anatomy, and readouts linked to sensorimotor behavior, including acoustic startle responsiveness, pain perception, and balance beam performance. This cross-species concordance suggests that these effects are particularly robust across species and that there is strong penetrance from the genotype to these phenotypes. Within the limitations discussed above, there is strong rationale to monitor the corresponding human versions of these readouts during clinical trials that entail chronic dosing of selective BACE1 inhibitors. Interestingly, data from early phase testing of BACE1 inhibitors in humans has begun to emerge. While the BACE1 inhibitors that were most recently tested in humans had a relatively benign side effect profile following acute treatment or daily treatment of up to 2 weeks^46^,^48^,^49^, clearly, the ultimate test of the safety of these compounds will be following long-term treatment. Based on the data presented here, it will be very interesting to specifically assess data from long-term trials in measures of sensorimotor function and nerve conduction velocity.

The interpretation of measures that diverge between rats and mice lacking BACE1 is more problematic. It would be valuable to know what factors account for the species differences in these measures. The *Bace1*^−/−^ rat model was established and maintained on the outbred Sprague-Dawley (Taconic) genetic background. In contrast, the *Bace1* targeted mouse allele of the mouse model used in the present study, was generated in 129 ES (R1) cells and subsequently backcrossed to C57BL/6J to establish a congenic strain^25^. It is possible that the increased genetic diversity in the rat versus the mouse can explain some of the discordant observations. From this perspective, the rat may offer a more translatable model, since the outbred line better reflects the genetic diversity in human populations. In addition, since the *Bace1* null mouse allele was generated in the context of the 129 strain, a number of closely linked genes from the original 129 strain that could modify the phenotype are likely to be still present in the *Bace1*^−/−^ mice but absent in *Bace1*^+/+^ littermates^51^. One published phenotypic difference between independently derived *Bace1*^−/−^ mouse lines supports the hypothesis that modifier genes from the background strain could lead to different phenotypes within the same species: May *et al*.^52^, also using the model of the present study, reported normal retinas in their *Bace1*^−/−^ mouse line. However, Cai *et al*.^23^ reported a thinning of the retina in their *Bace1*^−/−^ mouse line. The *Bace1* ko allele in their study was also generated in 129 ES cells, but then maintained on a mixed genetic background. It is now possible, using ZFN or CRISPR technology, to generate targeted alleles directly in a pure mouse background, allowing direct comparison of the *Bace1* null phenotype in pure 129 and C57BL/6J backgrounds to see if any of the mouse/rat discordances reported here can be explained by strain-specific modifier genes. In contrast, the influence of homozygous recessive modifier alleles is likely minimal in outbred rats. Existing mouse studies support a role of genetic background on the expression of *Bace1*^−/−^phenotypes. For example, Luo *et al*.^42^ developed one of the first *Bace1*^−/−^ mouse models. This model was on the outbred Black Swiss background and they did not find any overtly abnormal phenotype. Subsequently, other *Bace1*^−/−^ mouse models were developed on inbred background strains revealing *Bace1*^−/−^ phenotypes^12^,^13^,^18^,^19^,^20^,^21^. Overall, the observed *Bace1*^−/−^ phenotypic differences between mouse genetic backgrounds makes it difficult to completely separate the relative role of species and strain background in the present study and it is possible that differences in genetic background could, at least in part, have contributed to the apparent species differences. More studies are needed to evaluate this possibility. For example, one option may be the direct comparisons of congenic *Bace1*^−/−^ rat and mouse models that were both generated on an inbred background strain. However, no such rat model is available at present.

We have developed a novel rat line featuring BACE1 deletion, established several BACE1-dependent *in vivo* effects in this model, and compared those side by side with an established BACE1 knockout mouse model. This study broadens our understanding of BACE1 deletion across species, and suggests that some effects of BACE1 inhibition depend on the biological context (e.g. species, genetic background). The availability of genetic models in multiple species, coupled with preclinical pharmacological studies, offers the potential for much stronger predictive validity in determining the likely effects of prolonged BACE1 inhibition in humans.

## Additional Information

**How to cite this article:** Weber, M. *et al*. BACE1 across species: a comparison of the *in vivo* consequences of BACE1 deletion in mice and rats. *Sci. Rep.* **7**, 44249; doi: 10.1038/srep44249 (2017).

**Publisher's note:** Springer Nature remains neutral with regard to jurisdictional claims in published maps and institutional affiliations.

## Supplementary Material

Supplements

## Figures and Tables

**Table 1:** Summary of findings in *Bace1* ^−/−^ mice and rats relative to *Bace1* ^+/+^ controls.

Readout	Species		Figures	Mouse Studies
	Mouse	Rat		
Biochemistry				
BACE1 protein	(−) Absent	(−) Absent	1a–c	42,43
Aβ40 concentration (brain, plasma)	Not Tested	(↓) Reduced	1d–g	42
Basic health				
Overall mortality	(↑) Strongly Increased	(↑) Increased	2a,bS1a,b	12
Mortality, post-weaning	(↑) Increased	(0) No Change	2c,dS1c,d	12
Body weight, adult animals	(↓) Reduced	(0) No Change	3a,b	12,13
Behavior				
Spontaneous locomotor activity	(↑) Increased	(↓) Reduced	4a,b	12, 13, 14
Startle magnitude	(↓) Reduced	(↓) Reduced	5a,b	14
% PPI	(↓) Reduced	(↑) Increased	5c,dS2c,d	14
Olfactory function	(0) No deficit	(0) No deficit	6a–d	19, 20, 21^*^
Thermal pain response latency	(↑) Increased	(↑) Increased	7a,b	17 ^$^
Foot slips on balance beam	(↑) Increased	(↑) Increased	8a,b	18
Sciatic nerve anatomy				
Axon density	(↑) Increased	(↑) Increased	9b,c	16,17
Cross-sectional area of axon lumen	(↓) Reduced	(↑) Increased, not significant	9d,e	16,17
Area of myelin sheath surrounding axon	(↓) Reduced	(↓) Reduced	9f,g	16,17
g-ratio	(↑) Increased	(↑) Increased	9h,i	16,17

The summary table lists the effects of BACE1 deletion in mice and rats on readouts of biochemistry, basic health, behavior, and sciatic nerve anatomy. References to figures and corroborating mouse studies are included.

^*^Referenced studies investigated olfactory system formation but not olfactory function.

^$^Referenced study reports contrasting findings.
