# Supplementary material for: BACE1 across species: a comparison of the in vivo consequences of BACE1 deletion in mice and rats
Source: Sci Rep. 2017 Mar 10;7:44249. doi: 10.1038/srep44249 (PMC5345047; doi:10.1038/srep44249)
Supplement: Supplements [file srep44249-s1.pdf]

SUPPLEMENTS

BACE1 across species: a comparison of the *in vivo* consequences of BACE1 deletion in mice and rats

Martin Weber[1*&], Tiffany Wu[1&], William J. Meilandt[1], Sara L. Dominguez[1], Hilda O. Solanoy[1], Janice A. Maloney[1], Hai Ngu[2], Miriam Baca[2], Chung Kung[3], Lisa Lima[3], Timothy K. Earr[1], Daniel Fleck[1], Shannon D. Shields[1], William F. Forrest[4], Oded Foreman[2], Søren Warming[5], Ryan J. Watts[1], Kimberly Scearce-Levie[1]

[&] equal contribution

1. Department of Neuroscience, Genentech, 1 DNA Way, South San Francisco, CA 94080
2. Department of Pathology, Genentech, 1 DNA Way, South San Francisco, CA 94080
3. Department of Transgenic Technology, 1 DNA Way, South San Francisco, CA 94080
4. Department of Bioinformatics, 1 DNA Way, South San Francisco, CA 94080
5. Department of Molecular Biology, 1 DNA Way, South San Francisco, CA 94080

*Correspondence should be addressed to:

Martin Weber, PhD.

Department of Neuroscience, Genentech, 1 DNA Way, South San Francisco, CA 94080

Phone: +1-650-225-7951

Email: weber.martin@gene.com

SUPPLEMENTARY MATERIALS AND METHODS

Statistical Analysis

Startle and PPI data were analyzed using mixed-design or between-subjects ANOVAs. Mortality data from our animal breeding colonies was analyzed using several alternative scenarios via Kaplan-Meier survival estimates followed by generalized Wilcoxon Chi-Square (χ^2) tests for the estimated survival functions between genotypes. Results of survival analyses from animal data may potentially vary depending on how the analyses account for animals that were euthanized after a health alert had been issued. Thus, a scenario complementary to the one in the main paper was conducted here: In the main paper, the day that an animal with a previous health alert was euthanized is considered as the date at which the censoring criterion was met. This scenario takes advantage of the documented survival of the animal until the censoring date, but does not make any assumptions as to how long the animal may have lived after the censoring date. Here, an additional scenario was explored in which the day that an animal with a previous health alert was sacrificed is considered as the date of its death, implying that the animal would not have lived beyond the day it had been sacrificed. As in the model from the main paper, the day an animal was found dead or not found in the cage was considered the day of its death. As before in the main paper, censoring criteria was met on the day that an animal was 1) designated for transfer out of the breeding colony, or 2) alive on the last day that the database had been updated (4/4/2016). The data on animals that were flagged as runts (according to visual inspection by experienced colony managers) is based on animals that met any of these criteria on or before 28 days of age. The analyses of the runt data are based on Fisher's exact tests, given the nominal variable types ("genotype" and "runt vs. no runt") as well as the small number of events. Line or bar graphs with mean ± SEM values are shown. A 2-tailed alpha of 0.05 was used. * $p < 0.05$, ** $p < 0.01$, *** $p < 0.0001$.

Supplementary Results

Mortality in *Bace1*$^{-/-}$ animals is increased for both mice and rats relative to *Bace1*$^{+/-}$ and *Bace1*$^{+/+}$animals

Complementary to the analyses conducted in the main manuscript additional survival analyses were carried out that handle documented health alerts differently (see **supplementary analysis**). Kaplan-Meier survival estimates were conducted followed by generalized Wilcoxon Chi-Square tests for the estimated survival functions between genotypes based on a total of 6,010 mice (1567 +/+, 3314 +/-, and 1129 -/-). As in the main paper, these analyses revealed a genotype effect on mortality in mice, with higher mortality in *Bace1*$^{-/-}$ mice when compared to *Bace1*$^{+/+}$ mice as well as *Bace1*$^{+/-}$ mice, while no significant differences were found between +/+ and +/- mice (χ^2 (2) = 354.64 ($p < 0.0001$); +/+ < -/-, χ^2 (1) = 188.72 ($p < 0.0001$); +/- < -/-, χ^2 (1) = 278.95 ($p < 0.0001$); +/+ vs. +/-, χ^2 (1) = 0.73 ($p = 0.39$); **Figure S1a**).

Corresponding analyses in a total of 2,203 rats (422 +/+, 1380 +/-, and 401 -/-) also detected a genotype effect on mortality with higher mortality in *Bace1*$^{-/-}$ rats when compared to *Bace1*$^{+/+}$ rats as well as *Bace1*$^{+/-}$ rats, while no significant differences were found between +/+ and +/- rats (χ^2 (2) = 72.69 ($p < 0.0001$); +/+ < -/-, χ^2 (1) = 20.30 ($p < 0.0001$); +/- < -/-, χ^2 (1) = 62.37 ($p < 0.0001$); +/+ vs. +/-, χ^2 (1) = 0.20 ($p = 0.65$); **Figure S1b**).

Since mortality appeared most pronounced in young animals, subgroup analyses were again conducted in adult animals to assess if mortality in *Bace1*$^{-/-}$ animals remains increased in adulthood using model parameters distinct from the ones used in the main manuscript (see **supplementary methods** above). Juvenile animals that were dead or censored before the age of 29 days were excluded and the analyses were repeated. For mice, this subgroup contained 4,293 animals in total

(1,086 +/+, 2,333 +/-, 874 -/-). A genotype effect was again detected, with increased mortality in *Bace1*$^{-/-}$ mice relative to *Bace1*$^{+/+}$ mice and *Bace1*$^{+/-}$ mice, while +/+ were not significantly different from +/- mice (χ^2 (2) = 121.23 (p < 0.0001); +/+ < -/-, (χ^2 (1) = 60.99 (p < 0.0001); +/- < -/-, χ^2 (1) = 100.29 (p < 0.0001); +/+ vs. +/-, χ^2 (1) = 0.01 (p = 0.94), **Figure S1c).**

In rats, the corresponding subgroup contained 1,843 animals in total (386 WT +/+, 1,100 +/-, 357 -/-). Similar analyses did not reveal an effect of genotype even though mortality was numerically higher in *Bace1*$^{-/-}$ and *Bace1*$^{+/-}$ rats relative to +/+ controls (χ^2 (2) = 0.08 (p = 0.96), **Figure S1d**).

Taken together, this secondary set of analyses with distinct underlying assumptions lead to virtually identical results to the ones outlined in the main manuscript, showing that these conclusions are not limited to narrow model assumptions.

***Runts are more frequent in Bace1*$^{-/-}$ mice and rats during the pre-weaning phase**

Mortality is increased for both *Bace1*$^{-/-}$ mice and rats if animals from the pre-weaning phase are included in the analyses. In contrast, mortality is not significantly different for *Bace1*$^{-/-}$ rats relative to control rats during the post-weaning phase, while *Bace1*$^{-/-}$ mice of this age group continue to have increased mortality (**Figure 2** and **S1**). Further, adult *Bace1*$^{-/-}$ rats have normal body weights, again in contrast to adult *Bace1*$^{-/-}$ mice (**Figure 3**). These data raise the question if *Bace1*$^{-/-}$ mice and rats are also more likely to have low body weight during the pre-weaning phase. We were particularly interested in very small animals that are likely to have very low body weights possibly indicating fragile health. We therefore mined our in-house breeding database for pre-weaning animals (≤ 28 days of age) and counted the number of animals that were or were not flagged as runts. Fisher's exact test for

groups of animals from all genotypes (*Bace1*$^{+/+}$, *Bace1*$^{+/-}$, *Bace1*$^{-/-}$) revealed significant differences between the genotypes for both mice (p<0.0001) and rats (p=0.0002) with higher percentages of runts for *Bace1*$^{-/-}$ mice and rats relative to their respective control groups (**Supplementary Table S1**). Pairwise post-hoc tests revealed significant differences between *Bace1*$^{+/-}$ and *Bace1*$^{-/-}$ mice and between *Bace1*$^{+/+}$ and *Bace1*$^{-/-}$ mice (p<0.0001, each). The respective post-hoc tests for rats revealed significant differences between *Bace1*$^{+/-}$ and *Bace1*$^{-/-}$ rats (p=0.0003), but not between *Bace1*$^{+/+}$ and *Bace1*$^{-/-}$ rats (p=0.13). Of note, this difference in p-values between these two comparisons relative to *Bace1*$^{-/-}$ mice is driven by the different statistical power that is afforded by the different sample sizes in the *Bace1*$^{+/-}$ group (n=280) when compared to the *Bace1*$^{+/+}$ group (n=36) rather than the number of runts that were observed in these groups (0 runts for both *Bace1*$^{+/-}$ and *Bace1*$^{+/+}$ mice). Taken together, the higher fraction of *Bace1*$^{-/-}$ runts of both species during the pre-weaning phase suggests an increased likelihood for poor general health. These data corroborate the increased mortality found in *Bace1*$^{-/-}$ mice and rats of this age.

PPI is reduced in *Bace1*$^{-/-}$ mice and increased in *Bace1*$^{-/-}$ rats in subgroups that were matched for startle magnitude

To assess the impact of genotype effects on startle magnitude (see **Figure 5a,b**) on the PPI measures, separate analyses were carried out for subsets of animals constructed by eliminating the extreme responders until the effects of genotype on startle magnitude were numerically balanced across genotypes. For mice, mean (SEM) of startle magnitude in these subsets was: +/+: 43.18 (4.68); -/-: 39.83 (7.99); **Figure S2a**). ANOVA of %PPI in these subsets of mice again revealed the main effect of genotype with reduced %PPI for -/- mice, the expected effect of prepulse intensity with higher %PPI with higher prepulse intensities, but no interaction effect of genotype by prepulse intensity (genotype: $F(1,18) = 14.02$ ($p = 0.002$); prepulse intensity: ($F(2,36) = 28.57$ ($p < 0.0001$); genotype x prepulse intensity: $F(2,36) = 1.56$ ($p = 0.23$), **Figure S2c**).

Similar analyses were carried out for rats (mean (SEM) of startle magnitude in these subsets of rats: +/+: 65.24 (3.58); -/-: 62.79 (3.81); **Figure S2b**). ANOVA of %PPI in these subsets of rats again revealed the main effect of genotype with increased %PPI for -/- rats, the expected effect of prepulse intensity with higher %PPI for higher prepulse intensities, but no interaction effect of genotype by prepulse intensity ((genotype: $F(1,124) = 30.48$ ($p < 0.0001$); prepulse intensity: $F(2,248) = 187.83$ ($p < 0.0001$); genotype x prepulse intensity: $F(2,248) = 1.09$, ($p = 0.34$), **Figure S2d**).

These data confirm the results from the main paper and demonstrate in addition that the genotype effect on startle magnitude is unlikely to account for the genotype effect on %PPI for either mice or rats.

SUPPLEMENTARY Table Legend

Table S1. Overview of runts of pre-weaning age in our breeding colony data base.

The table lists the number of animals from both species and genotypes (*Bace1*$^{+/+}$, *Bace1*$^{+/-}$, *Bace1*$^{-/-}$ animals) of pre-weaning age (≤ 28 days) that were, or were not flagged as runts in the breeding colony database. The % of runts of each genotype is also shown for both mice and rats.

SUPPLEMENTARY Figure Legends

Figure S1. Effects of BACE1 deletion on survival in mice and rats.

(A) *Bace1*$^{-/-}$ mice (a) and rats (b) showed significantly increased mortality relative to *Bace1*$^{+/-}$ and *Bace1*$^{+/+}$ animals. Since a major fraction of the mortality occurred before or at weaning age, subgroup analyses were carried out in animals that lived beyond the weaning phase (≥ 29 days; c, d). In these subgroups of animals, *Bace1*$^{-/-}$ mice had increased mortality when compared to wildtype mice (c), while the corresponding subgroup of *Bace1*$^{-/-}$ rats were statistically indistinguishable from their wildtype counterparts (d) indicating that mortality is increased in *Bace1*$^{-/-}$ mice beyond the weaning age, but largely normal in *Bace1*$^{-/-}$ rats. ***$p < 0.0001$. Values shown represent the fraction of animals surviving. Numbers represent sample size (number of animals, n).

Figure S2. Effects of BACE1 deletion on %PPI in subgroups of mice and rats matched for startle magnitude between genotypes.

(**a, b**) Subgroups of animals that were matched for startle magnitude between genotypes. (**c, d**) In subgroups of animals that were matched for startle magnitude between genotypes %PPI was again significantly decreased in *Bace1*$^{-/-}$ mice, yet significantly enhanced in *Bace1*$^{-/-}$ rats, showing that the

genotype effects on startle magnitude is unlikely to account for the genotype effect on %PPI. ***p < 0.0001. Values are expressed as mean ± SEM, numbers represent sample size (n).

Table S1: Overview of runts ≤ 28 days of age in our breeding colony database

	Species							
	Mouse				Rat			
	**+/+**	**+/-**	**-/-**	Σ	**+/+**	**+/-**	**-/-**	Σ
# of normal-sized animals	470	959	189	1618	36	280	40	356
# of runts	11	22	66	99	0	0	4	4
Σ	481	981	255	1717	36	280	44	360
**% of runts**	**2.3**	**2.2**	**25.9**		**0**	**0**	**9.1**	

Figure S1

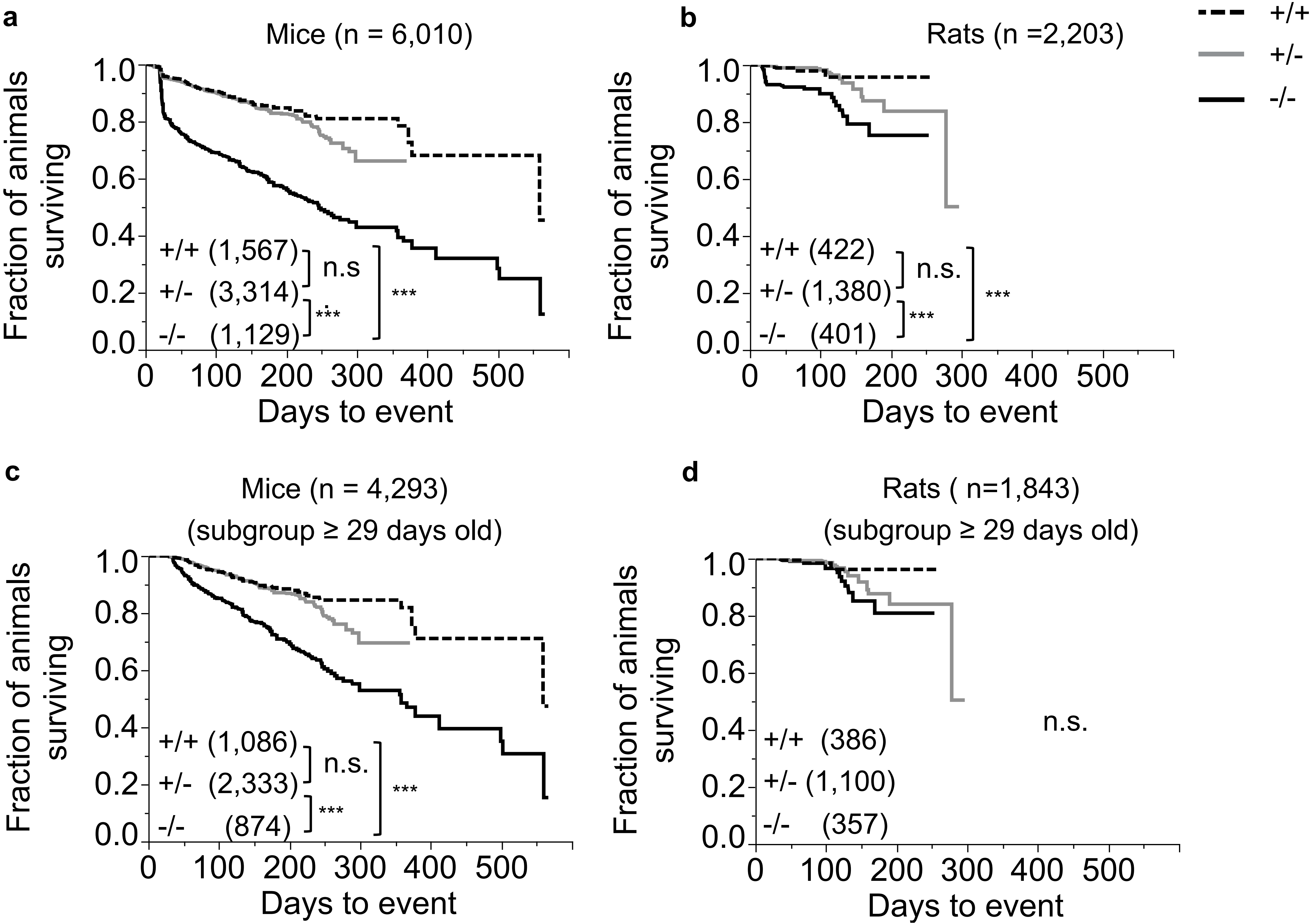

a
Mice (n = 6,010)
b
Rats (n =2,203)
+/+
+/-
-/-
Fraction of animals surviving
Days to event
+/+ (1,567)
+/- (3,314)
-/- (1,129)
n.s
***
***
+/+ (422)
+/- (1,380)
-/- (401)
n.s.
***
***
c
Mice (n = 4,293)
(subgroup ≥ 29 days old)
d
Rats (n=1,843)
(subgroup ≥ 29 days old)
+/+ (1,086)
+/- (2,333)
-/- (874)
n.s.
***
***
+/+ (386)
+/- (1,100)
-/- (357)
n.s.

Figure S2

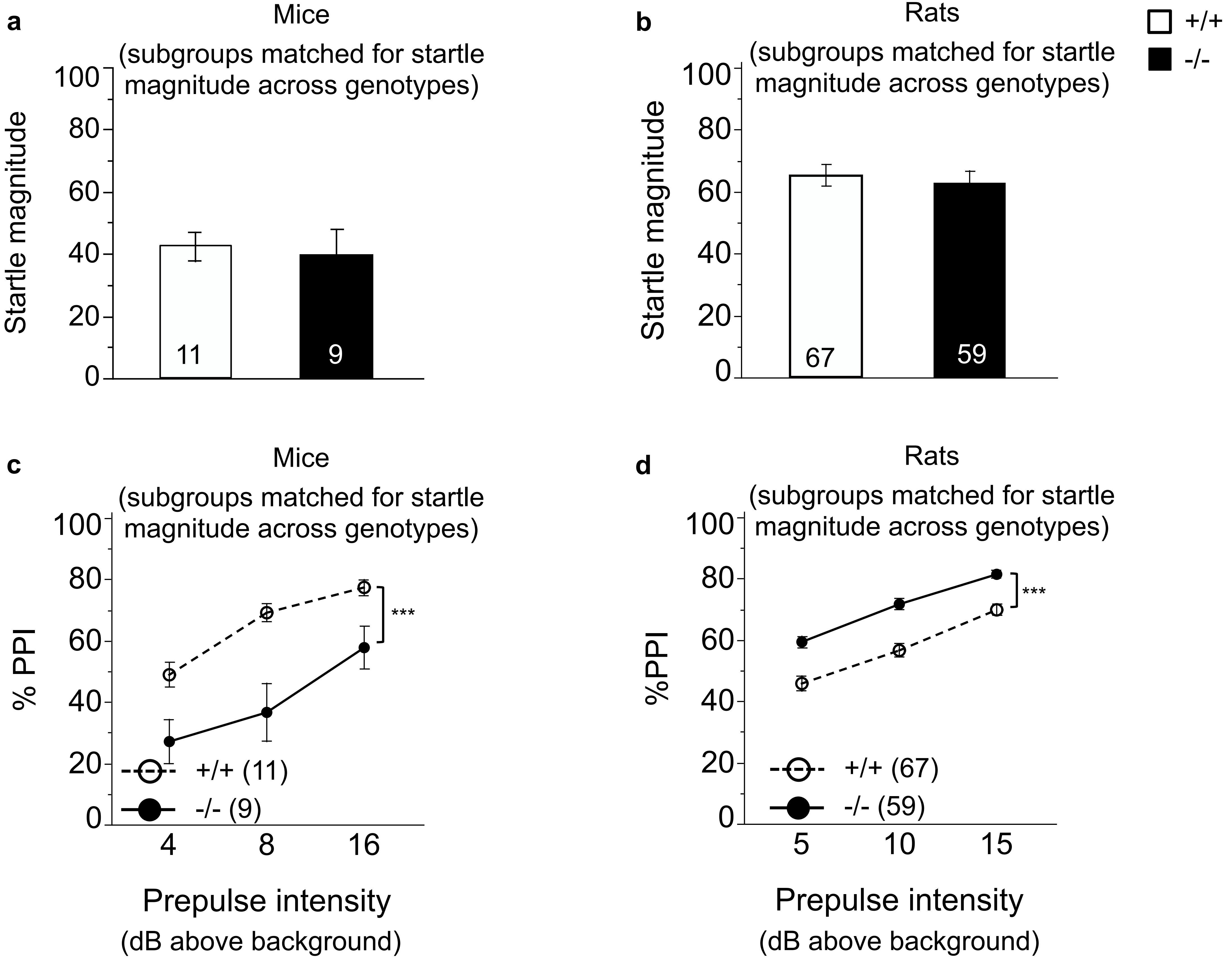
a
Mice
(subgroups matched for startle magnitude across genotypes)
Startle magnitude
0
20
40
60
80
100
11
9
b
Rats
(subgroups matched for startle magnitude across genotypes)
+/+
-/-
Startle magnitude
67
59
c
Mice
(subgroups matched for startle magnitude across genotypes)
% PPI
***
+/+ (11)
-/- (9)
4
8
16
Prepulse intensity
(dB above background)
d
Rats
(subgroups matched for startle magnitude across genotypes)
%PPI
***
+/+ (67)
-/- (59)
5
10
15
Prepulse intensity
(dB above background)
